# ALDH3B2 Polymorphism Is Associated with Colorectal Cancer Susceptibility

**DOI:** 10.1155/2020/5179635

**Published:** 2020-01-04

**Authors:** Zhi-Gang Gao, Yong Yang, Xiao-Feng Han, Yun-Lei Wang, Zhen-Jun Wang

**Affiliations:** Department of General Surgery, Beijing Chao-Yang Hospital, Capital Medical University, Beijing 100020, China

## Abstract

Colorectal cancer (CRC) is the 5^th^ leading cancer in China. Alcohol consumption has been reported to be one of the risk factors of CRC. However, it remains unclear whether genetic variants of alcohol metabolic genes are associated with CRC risk. In this study, we tested the coding variants in the alcohol metabolic genes and the risk of CRC, by using 485 cases and 516 controls. A total of 16 germline coding variants in 10 alcohol metabolic genes were genotyped. We identified that rs3741178 in ALDH3B2 was significantly associated with CRC risk with odds ratio being 2.13 (95% CI: 1.24–3.68, *P*=0.0064). Further functional annotation suggested that this variant may damage the protein function of ALDH3B2. Our results suggested that ALDH3B2 in the alcohol metabolism pathway contributed to the development of CRC, which may contribute to the prevention of this disease in the future.

## 1. Introduction

Colorectal cancer (CRC) ranks the fifth leading cause of cancer death among both men and women in China [1]. Epidemiology studies suggest that overnutrition, obesity, tobacco smoking, and heavy alcohol drinking are risk factors of this cancer [2]. Previous genome-wide association study (GWAS) has identified nearly 100 genetic loci that were associated with CRC susceptibility, mostly located in the noncoding region [3]. Recently, several exome-wide association studies identified that functional coding variants also played an important role in the susceptibility of multiple cancers [4–6]. Therefore, more variants, especially functional coding ones, still need to be further explored.

Alcohol drinking was an important risk factor for colorectal cancer [7]. Gene-drinking interaction was proved to be associated with risk of upper aerodigestive tract cancers, especially for esophageal cancer [8, 9]. Although a few studies found that genetic polymorphisms in the ALDH2 were associated with colorectal cancer risk [10–12], genetic variants in the alcohol metabolic genes have not been systematically explored for the susceptibility for this cancer.

In the present study, we searched for variants in the alcohol metabolic genes and performed a case-control study to test whether these variants are associated with CRC susceptibility. We found rs3741178 in ALDH3B2 was significantly associated with CRC risk. Further functional prediction found that this variant may damage the protein function of ALDH3B2.

## 2. Materials and Methods

### 2.1. Study Subjects

This study included 485 CRC patients and 516 healthy controls. The CRC cases were enrolled from Beijing Chao-Yang Hospital from January 1, 2016, to December 31, 2018. All cases were histopathologically or cytologically confirmed by at least two local pathologists according to the World Health Organization classification. Health controls were cancer-free individuals who lived in the same residential areas and were seeking for routine physical examination in the same time period from the same hospital where cases were collected. All participants were unrelated Han Chinese descent. The informed consent was obtained from every participant at recruitment, and peripheral blood samples and demographic characteristics such as gender, age, smoking status, drinking status, and ethnicity were collected by interviewers. This study was conducted under the approval of the Institutional Review Board of Beijing Chao-Yang Hospital, Capital Medical University.

### 2.2. SNP Selection and Genotyping

We searched for alcohol metabolic genes using Molecular Signature Database (http://www.broadinstitute.org/gsea/msigdb/index.jsp). We included only missense variants in alcohol metabolic genes (ADH and ALDH gene families) with minor allele frequencies (MAF) >0.01 in the Chinese Han population from the 1000 Genome Project. A total of 16 germline coding variants in 10 genes (ADH1C, ADH4, ADH7, ALDH1A1, ALDH1A2, ALDH1A3, ALDH1B1, ALDH3B1, ALDH3B2, and ALDH2) were selected and genotyped. Genotyping was performed using genomic DNA extracted from peripheral blood sample collected from each participant at recruitment. The DNA was extracted using TIANamp® Genomic DNA kit (Tiangen Biotech). SNPs were genotyped by the Sequenom MassARRAY system (San Diego, CA, USA). The case and control samples were mixed in the plates, and persons who performed the genotyping assay were not aware of case or control status.

### 2.3. Statistical Analysis

The association between SNPs and CRC risk was tested by using logistic regression analysis with adjustment of sex and age. Statistical analyses were performed using SPSS software (18.0). All tests were two-sided.

### 2.4. Functional Annotation Analysis

Multiple functional annotation tools were used to predict the potential function of rs3741178, including PolyPhen-2 [13], SIFT [14], and AWESOME [15].

## 3. Results

### 3.1. Characteristics of Study Subjects

The distributions of selected demographic characteristics including sex and age of the CRC patients and healthy controls are summarized in Table 1. This study consisted of 485 CRC cases and 516 controls. The average age of cases and controls was 61.23 (±12.47) and 61.68 (±11.81), respectively. There are 357 (73.6%) males and 128 (26.4%) females in the cases, while there are 358 (69.4%) males and 158 (30.6%) females in controls. Among cases, 188 (38.8%) and 138 (28.5%) were smokers and drinkers, respectively.

### 3.2. rs3741178 in ALDH3B2 Was Significantly Associated with CRC Risk

A total of 16 germline coding variants in the alcohol metabolic genes were genotyped, and their association with CRC risk was tested (Table 2). Among these variants, only rs3741178 in ALDH3B2 was significantly associated with increased risk of CRC with *FDR* <0.25 (Table 2). There were no significant gene-drinking interactions for these variants (Table 2). Compared with CC genotype carriers, CT genotype carriers were associated with CRC risk with odds ratio (OR) being 2.29 (95% CI: 1.26–4.14, *P*=0.0064) (Table 3). When combing the CT genotype and TT genotype carriers, the association was also significant with *P* values being 0.0050 (Table 3). There were no significant differences in the stratification analysis for this variant (Table 4).

### 3.3. rs3741178 in ALDH3B2 Was Predicted to Damage the Protein Function

The rs3741178 variant was located in the 2nd exon of *ALDH3B2* with Ala > Thr change. To test the potential function of rs3741178, we used multiple coding variant prediction tools. PolyPhen-2 showed that this variant was predicted to be “PROBABLY DAMAGING” with a score of 1.000, based on a number of features comprising the sequence, phylogenetic, and structural information characterizing the substitution. The SIFT result showed that this variant was predicted to be deleterious with the score being 0.009, indicating that the amino acid (rs3741178 Ala) was highly conserved in the protein family. The AWESOME showed that no significant posttranslational modification was affected by rs3741178. These results suggested that rs3741178 may damage the protein function of ALDH3B2 and thus associated with risk of colorectal cancer.

## 4. Discussion

In this case-control study, we explored the association between variants in the alcohol metabolic genes and colorectal cancer risk in 485 CRC cases and 516 controls. We found only rs3741178 in ALDH3B2 were significantly associated with an increased risk of CRC. Further functional prediction showed that this variant may damage the protein function of ALDH3B2. These results indicated an important role of ALDH3B2 in the CRC carcinogenesis.

ALDH3B2, also known as ALDH8, was a member of the aldehyde dehydrogenase family. This gene family, including ALDH1A1, ALDH1A2, ALDH1A3, ALDH2, ALDH3A1, ALDH3A2, ALDH3B1, and ALDH3B2, plays a major role in the detoxification of aldehydes generated by alcohol metabolism. ALDH1 and ALDH2 are the most important enzymes for aldehyde oxidation. ALDH1A1 and ALDH1B1 were shown to be markers for colorectal cancer [16, 17]. ALDH1A3 was reported to affect colon cancer proliferation and invasion [18]. The genetic polymorphisms in ALDH2 were reported to be associated with colorectal cancer [10–12].

However, little is known about ALDH3B2 in the development of cancer, especially CRC before this study. It was reported that DNA methylation of ALDH3B2 was associated with alcohol dependence [19]. Polymorphism in ALDH3B2 was reported to be associated with esophageal squamous cell carcinoma in a Chinese population [20]. In this study, we found that the polymorphism of ALDH3B2 was associated with CRC risk also in a Chinese population. These results indicated that ALDH3B2 may play an important role in the carcinogenesis of CRC by altering the alcohol metabolism process. The function of this gene in the development of CRC is worth to be investigated. Furthermore, rs3741178 variant may serve as a potential marker for the early detection of CRC.

There are also some limitations for this study. First, our sample size has only 24%–78% power to identify variants with OR being 2.0 with MAF = 0.01 to 0.05, indicating that the statistical power of this study may be insufficient. The results need to be validated in larger samples in the future. Second, the sample size was too small to perform a gene-drinking interaction with sufficient power. The interaction between these variants and drinking in the susceptibility of CRC needs to be investigated in the future. Finally, the function of ALDH3B2 variant was only predicted in this study. In vitro and in vivo experiments need to be conducted to validate the prediction results.

In summary, through a case-control study in a Chinese Han population, we find a significant association between the coding variant in ALDH3B2 and CRC risk. These results expand our insights of CRC carcinogenesis and provide more evidence for the precision medicine of this disease.

## Figures and Tables

**Table 1:** Summary of characteristics of study subjects.

	Cases (*n* = 485)	Controls (*n* = 516)	*P* ^a^
Age (years), mean ± S.D.	61.23 ± 12.47	61.68 ± 11.81	
Gender, *n* (%)			0.1389
Male	357 (73.6)	358 (69.4)	
Female	128 (26.4)	158 (30.6)	
Smoking status			0.1613
Nonsmoker	297 (61.2)	338 (65.5)	
Smoker	188 (38.8)	178 (34.5)	
Drinking status			0.1686
Nondrinker	347 (71.5)	389 (75.4)	
Drinker	138 (28.5)	127 (24.6)	

^a^Calculated by the Chi-squared test.

**Table 2:** Genetic variants in the alcohol metabolism genes and their association with CRC risk.

Chr	SNP	Position	Gene	Location	Effect allele	MAF cases	MAF controls	*P* ^a^	OR (95% CI)^b^	*P* ^b^	*FDR*	*P* ^c^
4	rs55735703	100057727	ADH4	Missense	A	0.01	0.01	1.0000	0.70 (0.31–1.58)	0.3945	0.7976	0.3830
4	rs698	100260789	ADH1C	Missense	G	0.08	0.09	0.5748	0.92 (0.67–1.27)	0.6049	0.8483	0.0664
4	rs1693482	100263965	ADH1C	Missense	A	0.08	0.09	0.7793	0.92 (0.66–1.27)	0.6117	0.8483	0.0645
4	rs971074	100341861	ADH7	Synonymous	A	0.11	0.13	1.0000	0.82 (0.63–1.07)	0.1429	0.6576	0.7415
9	rs2228093	38396002	ALDH1B1	Missense	A	0.37	0.39	0.7109	0.90 (0.75–1.09)	0.2769	0.7976	0.0895
9	rs2073478	38396065	ALDH1B1	Missense	A	0.31	0.32	1.0000	0.97 (0.80–1.17)	0.7128	0.8773	0.0368
9	rs113083991	38396271	ALDH1B1	Missense	A	0.02	0.03	0.3328	0.82 (0.47–1.44)	0.4875	0.8483	0.6004
9	rs8187929	75540504	ALDH1A1	Missense	A	0.03	0.04	0.5069	0.78 (0.48–1.27)	0.3153	0.7976	0.5290
11	rs58465018	67430748	ALDH3B2	Missense	G	0.03	0.03	0.3521	1.05 (0.63–1.76)	0.8450	0.9013	0.7338
11	rs1551886	67430762	ALDH3B2	Missense	A	0.09	0.09	0.2544	1.00 (0.74–1.37)	0.9753	0.9753	0.6181
**11**	**rs3741178**	**67434048**	**ALDH3B2**	**Missense**	**T**	**0.04**	**0.02**	0.2544	**2.13 (1.24–3.68)**	**0.0064**	**0.1024**	**0.7677**
11	rs308341	67795299	ALDH3B1	Missense	A	0.26	0.27	0.1553	0.95 (0.78–1.17)	0.6362	0.8483	0.4126
11	rs3751082	67795353	ALDH3B1	Missense	A	0.26	0.27	0.0261	0.92 (0.75–1.12)	0.3988	0.7976	0.4694
12	rs671	112241766	ALDH2	Missense	A	0.28	0.25	0.0199	1.15 (0.94–1.41)	0.1642	0.6576	0.1748
15	rs4646626	58256127	ALDH1A2	Missense	A	0.31	0.31	1.0000	0.98 (0.81–1.18)	0.8123	0.9013	0.3010
15	rs3803430	101445815	ALDH1A3	Missense	G	0.05	0.06	0.2871	0.76 (0.52–1.12)	0.1644	0.6576	0.0916

Note: SNP, single nucleotide polymorphism; MAF, minor allele frequency. ^a^Test for the Hardy–Weinberg equilibrium in the control samples. ^b^Calculated by the logistic regression model adjusted for sex, age, smoking status, and drinking status. ^c^Calculated by the logistic regression model.

**Table 3:** Association between rs3741178 and risk of CRC.

SNP	Genotype	Number of cases (%)	Number of controls (%)	OR (95% CI)^a^	*P* ^a^
rs3741178	CC	448 (92.4)	498 (96.5)	1.00 (reference)	
	CT	35 (7.2)	17 (3.3)	2.29 (1.26–4.14)	0.0064
	TT	2 (0.0)	1 (0.0)	2.25 (0.20–25.05)	0.5086
	CT + TT	37 (7.6)	18 (3.5)	2.29 (1.28–4.08)	0.0050

^a^Calculated by the logistic regression model adjusted for sex, age, smoking status, and drinking status.

**Table 4:** Stratified analysis for rs3741178 and risk of CRC.

SNP	Stratification	OR (95% CI)^a^	*P* ^a^
rs3741178	Male	2.33 (1.19–4.58)	0.0138
	Female	1.33 (0.50–3.54)	0.5599
	>60 years	2.02 (0.95–4.30)	0.0659
	≤60 years	1.93 (0.85–4.39)	0.1187
	Nonsmoker	2.59 (0.98–6.87)	0.0554
	Smoker	1.85 (0.95–3.57)	0.0685
	Nondrinker	2.13 (0.68–6.69)	0.1947
	Drinker	1.99 (1.06–3.71)	0.0311

^a^Calculated by the logistic regression model adjusted for sex, age, smoking status, and drinking status.

## Data Availability

The data used to support the findings of this study are available from the corresponding author upon request.

## References

[1] Chen W., Zheng R., Baade P. D. (2016). Cancer statistics in China, 2015. *CA: A Cancer Journal for Clinicians*.

[2] Song M., Garrett W. S., Chan A. T. (2015). Nutrients, foods, and colorectal cancer prevention. *Gastroenterology*.

[3] Huyghe J. R., Bien S. A., Harrison T. A. (2019). Discovery of common and rare genetic risk variants for colorectal cancer. *Nature Genetics*.

[4] Chang J., Zhong R., Tian J. (2018). Exome-wide analyses identify low-frequency variant in CYP26B1 and additional coding variants associated with esophageal squamous cell carcinoma. *Nature Genetics*.

[5] Chang J., Tian J., Yang Y. (2018). A rare missense variant in TCF7L2 associates with colorectal cancer risk by interacting with a GWAS-identified regulatory variant in the MYC enhancer. *Cancer Research*.

[6] Chang J., Tian J., Zhu Y. (2018). Exome-wide analysis identifies three low-frequency missense variants associated with pancreatic cancer risk in Chinese populations. *Nature Communications*.

[7] Jayasekara H., MacInnis R. J., Room R., English D. R. (2016). Long-term alcohol consumption and breast, upper aero-digestive tract and colorectal cancer risk: a systematic Review and meta-analysis. *Alcohol and Alcoholism*.

[8] Wu C., Kraft P., Zhai K. (2012). Genome-wide association analyses of esophageal squamous cell carcinoma in Chinese identify multiple susceptibility loci and gene-environment interactions. *Nature Genetics*.

[9] Chang J., Tan W., Ling Z. (2017). Genomic analysis of oesophageal squamous-cell carcinoma identifies alcohol drinking-related mutation signature and genomic alterations. *Nature Communications*.

[10] Yang H., Zhou Y., Zhou Z. (2009). A novel polymorphism rs1329149 of CYP2E1 and a known polymorphism rs671 of ALDH2 of alcohol metabolizing enzymes are associated with colorectal cancer in a southwestern Chinese population. *Cancer Epidemiology Biomarkers and Prevention*.

[11] Zhong Q., Wu R. R., Zeng Z. M. (2016). Association of ADH1B Arg47His and ALDH2 Glu487Lys polymorphisms with risk of colorectal cancer and their interaction with environmental factors in a Chinese population. *Genetics and Molecular Research*.

[12] Li R., Zhao Z., Sun M., Luo J., Xiao Y. (2016). ALDH2 gene polymorphism in different types of cancers and its clinical significance. *Life Sciences*.

[13] Adzhubei I. A., Schmidt S., Peshkin L. (2010). A method and server for predicting damaging missense mutations. *Nature Methods*.

[14] Kumar P., Henikoff S., Ng P. C. (2009). Predicting the effects of coding non-synonymous variants on protein function using the SIFT algorithm. *Nature Protocols*.

[15] Yang Y., Peng X., Ying P. (2019). AWESOME: a database of SNPs that affect protein post-translational modifications. *Nucleic Acids Research*.

[16] Yang W., Wang Y., Wang W., Chen Z., Bai G. (2018). Expression of aldehyde dehydrogenase 1A1 (ALDH1A1) as a prognostic biomarker in colorectal cancer using immunohistochemistry. *Medical Science Monitor*.

[17] Matsumoto A., Arcaroli J., Chen Y. (2017). Aldehyde dehydrogenase 1B1: a novel immunohistological marker for colorectal cancer. *British Journal of Cancer*.

[18] Feng H., Liu Y., Bian X., Zhou F., Liu Y. (2018). ALDH1A3 affects colon cancer in vitro proliferation and invasion depending on CXCR4 status. *British Journal of Cancer*.

[19] Zhang R., Miao Q., Wang C. (2013). Genome-wide DNA methylation analysis in alcohol dependence. *Addiction Biology*.

[20] Yin J., Tang W., Long T. (2017). Association of ALDH3B2 gene polymorphism and risk factors with susceptibility of esophageal squamous cell carcinoma in a Chinese population: a case-control study involving 2,358 subjects. *Oncotarget*.

